# Resolving Tumor Heterogeneity: Genes Involved in Chordoma Cell Development Identified by Low-Template Analysis of Morphologically Distinct Cells

**DOI:** 10.1371/journal.pone.0087663

**Published:** 2014-02-04

**Authors:** Amin El-Heliebi, Thomas Kroneis, Karin Wagner, Katharina Meditz, Dagmar Kolb, Julia Feichtinger, Gerhard G. Thallinger, Franz Quehenberger, Bernadette Liegl-Atzwanger, Beate Rinner

**Affiliations:** 1 Institute of Pathology, Medical University of Graz, Graz, Austria; 2 Institute of Cell Biology, Histology & Embryology, Medical University of Graz, Graz, Austria; 3 Center for Medical Research, Medical University of Graz, Graz, Austria; 4 Institute for Genomics and Bioinformatics, Graz University of Technology, Graz, Austria; 5 Core Facility Bioinformatics, Austrian Centre of Industrial Biotechnology, Graz, Austria; 6 Institute for Medical Informatics, Statistics and Documentation, Medical University of Graz, Graz, Austria; University of Navarra, Spain

## Abstract

The classical sacrococcygeal chordoma tumor presents with a typical morphology of lobulated myxoid tumor tissue with cords, strands and nests of tumor cells. The population of cells consists of small non-vacuolated cells, intermediate cells with a wide range of vacuolization and large heavily vacuolated (physaliferous) cells. To date analysis was only performed on bulk tumor mass because of its rare incidence, lack of suited model systems and technical limitations thereby neglecting its heterogeneous composition. We intended to clarify whether the observed cell types are derived from genetically distinct clones or represent different phenotypes. Furthermore, we aimed at elucidating the differences between small non-vacuolated and large physaliferous cells on the genomic and transcriptomic level. Phenotype-specific analyses of small non-vacuolated and large physaliferous cells in two independent chordoma cell lines yielded four candidate genes involved in chordoma cell development. *UCHL3*, coding for an ubiquitin hydrolase, was found to be over-expressed in the large physaliferous cell phenotype of MUG-Chor1 (18.7-fold) and U-CH1 (3.7-fold) cells. The mannosyltransferase *ALG11* (695-fold) and the phosphatase subunit *PPP2CB* (18.6-fold) were found to be up-regulated in large physaliferous MUG-Chor1 cells showing a similar trend in U-CH1 cells. *TMEM144*, an orphan 10-transmembrane family receptor, yielded contradictory data as cDNA microarray analysis showed up- but RT-qPCR data down-regulation in large physaliferous MUG-Chor1 cells. Isolation of few but morphologically identical cells allowed us to overcome the limitations of bulk analysis in chordoma research. We identified the different chordoma cell phenotypes to be part of a developmental process and discovered new genes linked to chordoma cell development representing potential targets for further research in chordoma tumor biology.

## Introduction

Chordomas are malignant tumors, arise within the bones of the axial skeleton and show a destructive growth, with a phenotype that recapitulates the notochord [1], [2]. Chordoma tumors are comprised of morphologically heterogeneous cells, i.e. ranging from smaller non-vacuolated spindly shaped cells to large cells with prominent vacuoles, usually referred to as “physaliferous cells” [3]. Within the range of these two phenotypes exists a continuum of intermediate cells with various degrees of vacuolization [3]. There is evidence that the small cells but not the physaliferous cells are the proliferating cell population [4], [5].

Until today, the underlying molecular mechanisms for this high degree of heterogeneity within chordoma tumors has not been investigated, although holding great potential in revealing new drug targets. Thus, chemoresistancy of chordoma tumors may be due to its heterogeneity, whereby the slow or non-dividing cells escape chemotherapeutic treatment [6].

The phenotypic diversity in chordoma tissue is well reflected in recent established cell lines [7], [8]. In our study, we used the sacrococcygeal chordoma derived MUG-Chor1 cell line [8] that reflects classical chordoma tumor characteristics to analyze chordoma tumor heterogeneity. We performed laser capture microdissection and micromanipulation to obtain homogeneous cell populations of the small and the large physaliferous cell phenotypes in order to investigate their genomic as well as transcriptomic differences. Additionally, this study was conducted to elucidate the dynamic development and proliferation of chordoma cell phenotypes *in vitro*.

For the first time morphologically distinct cell phenotypes were separated and analyzed by means of molecular genetics and transcriptomics. Thereby we overcome the major drawback of bulk analyses to find differences within chordoma cell phenotypes allowing a deeper insight into chordoma tumor biology.

## Materials and Methods

### Cell Culture

MUG-Chor1 chordoma cell line cells (passage 30) established from a classic sacrococcygeal chordoma from a 58 year old Caucasian female [8] were cultured in IMDM/RPMI 4∶1 (PAA Laboratories, Pasching, Austria) supplemented with 2 mM L-Glutamine (PAA), 10% FBS (PAA) and 1% PS (PAA) at 37°C and 5% CO_2_. Culture medium was changed twice a week and splitting of the cell culture was done every ten days at confluency of 70–80%. Cells were kept in a 5% CO_2_ atmosphere at 37°C and periodically checked for mycoplasma by PCR. The cells were verified to be MUG-Chor1 by STR analysis using PowerPlex 16 System Kit (Promega, Mannheim, Germany).

U-CH1 chordoma cell line cells were kindly provided by Silke Brüderlein (Ulm University, Germany). The cells were cultured following the protocols for the MUG-Chor1 cells.

### Tumor Tissue

Formalin-fixed paraffin-embedded (FFPE) tissues were sectioned (4 µm) and forwarded to either hematoxylin and eosin (HE) or immunohistochemical staining using antibodies against brachyury (clone H-210; Santa Cruz, Santa Cruz, USA), pan-cytokeratin (clone MNF116, Dako), epithelial membrane antigen (EMA; clone E29, Dako) and S100 (polyclonal; Dako, Glostrup, Denmark). Tissue sections were subjected to antigen retrieval in a tris-borate/EDTA buffer (CC1, Ventana, Roche, Vienna, Austria) before incubating with clone H-210 (Santa Cruz, dilution 1∶50) for brachyury immunohistochemistry. Antibody detection was performed on a Ventana Immunostainer using the ultraView Universal DAB Detection Kit (Roche) according to the manufacturer’s recommendations.

### Electron Microscopy – Chemical Fixation

MUG-Chor1 cells grown on a Aclar film (Gröpl, Tulln, Austria) were fixed in 2.5% (wt/vol) glutaraldehyde (Agar Scientific Ltd., Stansted/Essex, UK) and 2% (wt/vol) paraformaldehyde (Merck KGaA, Darmstadt, Germany) in 0.1 M phosphate buffer, pH 7.4, for 2 h, postfixed in 2% (wt/vol) osmium tetroxide (Electron Microscopy Sciences, Hatfield, USA) for 2 h at room temperature, dehydrated in graded series of ethanol and embedded in a TAAB epoxy resin (Agar Scientific Ltd.). Ultrathin sections (70 nm thick) were cut with a Leica UC 7 Ultramicrotome (Leica Microsystems, Wetzlar, Germany) and stained with 2% lead citrate (Laurylab, St.Fons, France) for 5 min and with 0.5% uranyl acetate (Laurylab) for 15 min. Images were taken using a FEI Tecnai G2 20 transmission electron microscope (FEI company, Eindhoven, The Netherlands) with a Gatan ultrascan 1000 CCD camera (Gatan GmbH, Munich, Germany). Acceleration voltage was 120 kV.

### Laser Capture Microdissection and Whole Genome Amplification (WGA)

MUG-Chor1 cells were trypsinized (0.05% Trypsin-EDTA) and cytocentrifuged onto polyethylene naphthalate (PEN) membrane coated slides (Carl Zeiss GmbH, Vienna, Austria) as described previously [9]. Whole genome amplification (WGA) was performed using the GenomePlex Single Cell Whole Genome Amplification Kit (WGA4, Sigma-Aldrich, St. Louis, USA) as previously described [10] with slight modifications. In short, we catapulted 100 large physaliferous cells and 100 small non-vacuolated cells each into the lids of PCR-reaction tubes which already contained 10 µl of Single Cell Lysis and Fragmentation Buffer (WGA4). After cell lysis, fragmentation and GenomePlex library preparation, the samples were amplified by adding 61 µl consisting of 7.5 µl of 10x amplification master mix, 48.5 µl nuclease free water, and 5 µl WGA DNA polymerase. The amplified DNA was purified using the GenElute PCR Clean-Up Kit (Sigma-Aldrich). The purity and concentration was measured by a NanoDrop ND-1000 spectrophotometer (Thermo Fisher Scientific, Waltham, USA). The quality of amplified DNA was additionally assessed by a multiplex PCR as previously described [11].

### Array-CGH

Array-CGH analysis of MUG-Chor1 samples was performed using SurePrint G3 Human CGH Microarrays 8×60K (Agilent Technologies, Santa Clara, USA) with the Bioprime Array CGH Genomic Labeling System (Life Technologies, Carlsbad, USA) according to the manufacturers' manual. In brief, 250 ng of both WGA4 amplified sample and WGA4 amplified reference DNA were differentially labeled with dCTP-Cy5 and dCTP-Cy3 (GE Healthcare, Little Chalfont, UK), respectively. Subsequently, DNA was purified with Amicon Ultracel-30 filters (Millipore, Billerica, USA) and simultaneously hybridized onto the 60 k microarray slides at 65°C for 24 h using the Oligo aCGH/ChIP-on-chip Hybridization kit (Agilent Technologies). After hybridization, the arrays were washed and scanned (Agilent Technologies) as recommended by the manufacturer. The data was analyzed with Agilent Genomic Workbench Lite Edition 6.5.0.18 (Agilent Technologies). Software settings for analysis were as follows: ADM-2 algorithm, threshold 6.5, with at least three consecutive oligos and with an absolute log ratio of 0.35. Fuzzy zero was switched off and centralization was set to the threshold 6.0 with a bin size of 10.

### Whole Transcriptome Amplification (WTA)

MUG-Chor1 and U-CH1 cells were harvested by trypsinization and forwarded to micromanipulation on an inverted microscope (Zeiss Axiovert M 200) equipped with a micromanipulator (MMJ, Zeiss; CellTram vario, Eppendorf, Hamburg, Germany) using microcapillaries having an inner diameter of 20 µm (TransferTip, Eppendorf). Cell pools of 20 large, physaliferous cells and of 20 small cells were picked in quadruplicates each. WTA was performed with the WT-Ovation™ One-Direct RNA Amplification System (NuGEN Technologies, San Carlos, USA) as described in the user guide. Following WTA, amplified cDNA was purified using the MinElute Reaction Cleanup Kit (Qiagen, Hilden, Germany) and quantified by a NanoDrop ND-1000 (Thermo Fisher Scientific). The size distribution of amplified cDNA was analyzed on a Bioanalyzer BA2100 (Agilent Technologies) using the RNA 6000 Nano LabChip (Agilent Technologies).

For microarray analysis (MUG-Chor1 cells only) 5 µg of amplified cDNA were fragmented and labeled using the NuGEN Encore™ Biotin Module (NuGEN Technologies) according to the manual. The labeled MUG-Chor1 cDNA was hybridized to Affymetrix GeneChip Human 1.0 ST arrays (Affymetrix, Santa Clara, USA). Hybridization time was set to 45°C for 40 h while rotating in a hybridization oven as recommended. Washing and staining was performed with GeneChip® HT hybridization Wash and Stain Kit (Affymetrix) on the Affymetrix GeneChip® fluidics station 450 according to the manual. The arrays were scanned with Affymetrix GeneChip Scanner GCS3000. The MUG-Chor1 microarray data have been submitted to Gene Expression Omnibus (GEO, accession number: GSE48779).

### Low-template Microarray Analysis and Quantitative Real Time-PCR (RT-qPCR)

The MUG-Chor1 data has been pre-processed and analyzed in R 2.15.2 [12]. After quality control the raw data has been pre-processed according to methods described by Irizarry *et al.* [13], [14] using the ‘affy’ R package from Bioconductor [15]. The pre-processed data is available from GEO (GSE48779). To increase the statistical power, genes that were not expressed in the two phenotypes as well as genes showing no expression changes across all samples have been removed as described by Scholtens and von Heydebreck [16]. To compute differentially expressed genes between the two phenotypes, the ‘Limma’ R package from Bioconductor [17] was used. The p-values were adjusted for multiple testing with Benjamini and Hochberg's method to control the false discovery rate [18]. Genes with an adjusted p-value <0.05 and a |log2-fold change| >1 have been considered as significantly differentially expressed.

Furthermore, a self-contained gene set test, GlobalTest has been employed to the MUG-Chor1 data to detect differentially expressed sets of related genes using the ‘globaltest' R package from Bioconductor [19]. Available gene sets associated with gene ontology (GO) terms as well as all curated datasets available in MSigDB [20] have been tested for differential expression. All p-values were adjusted for multiple testing using Benjamini and Hochberg's method [18]. GO term associated and curated MSigDB gene sets with an adjusted p-value <0.05 have been considered as potentially significant.

We performed quantitative real-time PCR (RT-qPCR) of four genes (*ALG11*, *PPP2CB*, *TMEM144*, *UCHL3*), which were detected to be differentially expressed in MUG-Chor1 small cells compared to large cells by Affymetrix gene expression arrays and subsequent data analysis. Glyceraldehyde-3-phosphate dehydrogenase (*GAPDH*) and β-actin (*ACTB*) were used as housekeeping genes. Using the MUG-Chor1 cDNA we additionally run five genes [cytokeratins 8 (*KRT8*) and 19 (*KRT19*), brachyury (*T*), transforming growth factor α (*TGFA*), vimentin (*VIM*)] typically expressed in chordoma to assess their expression level in RT-qPCR analysis. We used amplified MUG-Chor1 cDNA of the picked small and large phenotypes with commercial available and pre-optimized TaqMan assays (Table 1) and TaqMan Gene Expression MasterMix (Applied Biosystems). All assays were run on an ABI 7900 Sequence Detection System (Applied Biosystems) using SDS 4.0 Software (Applied Biosystems) according to standard conditions. All samples were run in four biological and each in three technical replicates. Raw cycle threshold (Cq) values were tested for outlying values in R 2.15.2 [12]. Potential outliers were identified based on a box and whisker plot and were subsequently assessed using the Grubbs' test as described by Burns *et al.* [21]. Outliers with a p-value <0.05 have been removed. Mean ΔCq values were normalized to *ACTB* and *GAPDH*. Normalization and statistical analysis was done with GenEx Professional (MultiD Analysis Version 5.3.5.6, Gothenburg, Sweden) using t-test (unpaired, two-sided) followed by Dunn-Bonferroni *post hoc* comparison testing (cut-off for multiple testing was p = 0.01274).

**Table 1:** Expression analyses of chordoma specific and candidate genes in MUG-Chor1 cells.

Gene	TaqMan GeneExpression Assay	Mean Cq andstandard deviation^a^		Fold change^b^	p-value
		small cells	large cell	large vs. small	
*ALG11* c	Hs01076287_m1	34.9±1.7	26.6±0.7	695	1.4•10^−4^
*UCHL3*	Hs00234683_m1	31.0±1.3	27.6±0.4	18.8	1.9•10^−5^
*TMEM144*	Hs00938021_m1	29.7±0.6	31.6±0.3	−2.4	0.0061
*PPP2CB*	Hs00602137_m1	32.0±1.9	28.6±0.7	18.6	0.0016
*ACTB*	Hs01060665_g1	25.2±1.5	25.8±1.1	–	–
*GAPDH*	Hs02758991_g1	22.7±0.5	23.6±0.2	–	–
*VIM*	Hs00185584_m1	21.9±0.8	21.5±0.7	–	–
*T*	Hs00610080_m1	28.5±2.2	27.6±0.8	–	–
*KRT8*	Hs01595539_g1	28.5±0.6	27.9±0.3	–	–
*KRT19*	Hs00761767_s1	26.8±1.2	29.6±0.4	–	–
*TGFα*	Hs00608187_m1	27.7±0.9	27.2±0.4	–	–

RT-qPCR was done on AB7900 TaqMan (Applied Biosystems; Foster City, CA). *GAPDH* and *ACTB* were used for normalization. Normalization and statistical analysis was done with GenEx Professional (MultiD Analysis; Version 5.3.5.6). All non-template controls were undetermined (Cq>45) except for *GAPDH* showing two replicates with Cq values >37 and *VIM* yielding one replicate at Cq = 27. Cut-off for multiple testing (*ALG11*, *UCHL3*, *TMEM144* and *PPP2CB*) was p = 0.01274.

a calculated as mean values from quadruplicate or triplicate (in case the Cq value could not be defined) biological samples.

b Cq values were normalized to *GAPDH* and *ACTB* (ΔCq). Differential expression (ΔΔCq) is given as positive (up-regulated in large cells) or negative (down-regulated in large cells) fold change ( = 2^ΔΔCq^).

c Cq values w/o outlier. Outliers were identified by means of Grubbs’ outlier test.

For verifying the data obtained from MUG-Chor1 cells we isolated small and physaliferous U-CH1 cells following exactly the same procedures as for the MUG-Chor1 cells. The amplified U-CH1 cDNA was subjected to RT-qPCR analysis of *ALG11*, *PPP2CB*, *TMEM144* and *UCHL3* according to the settings as described for MUG-Chor1 cells (Table 2).

**Table 2:** Expression analyses of MUG-Chor1 candidate genes in U-CH1 cells.

Gene	TaqMan GeneExpression Assay	Mean Cq andstandard deviation^a^		Fold change^b^	p-value
		small cells	large cell	large vs. small	
*ALG11*	Hs01076287_m1	32.5±4.1	29.3±0.8	10.6	0.030
*UCHL3*	Hs00234683_m1	29.6±1.4	27.9±0.8	3.7	2.3•10^−4^
*TMEM144*	Hs00938021_m1	32.4±2.9	31.5±1.6	2.1	0.26
*PPP2CB*	Hs00602137_m1	33.4±4.5	31.3±1.5	4.9	0.026
*ACTB*	Hs01060665_g1	27.4±2.3	27.3±1.0	–	–
*GAPDH*	Hs02758991_g1	27.4±2.9	27.0±1.1	–	–

RT-qPCR was done on AB7900 TaqMan (Applied Biosystems; Foster City, CA). Normalization (*GAPDH* and *ACTB*) and statistical analysis was done with GenEx Professional (MultiD Analysis; Version 5.3.5.6; see also 2.7). Cut-off for multiple testing (*ALG11*, *UCHL3*, *TMEM144* and *PPP2CB*) was p = 0.01274.

a calculated as mean values from quadruplicate or triplicate (in case the Cq value could not be defined) biological samples.

b Cq values were normalized to *GAPDH* and *ACTB* (ΔCq). Differential expression (ΔΔCq) is given as positive (up-regulated in large cells) or negative (down-regulated in large cells) fold change ( = 2^ΔΔCq^).

### Cell Imaging (Cell-IQ) and Morphological Observations

The viability of MUG-Chor1 cells was assessed with a Casy Cell Counter Model TT (Roche). We seeded 4.0•10^5^ cells in 3 ml into each well of a 6-well plate (Nunc, Sigma Aldrich, Munich, Germany). Cell monitoring was done over seven days on the Cell-IQ V2 MLF (Chipman, Tampere, Finland) and images of cells were taken using a 10X objective (Nikon, Tokyo, Japan) every 30 min (Video S1).

We classified cells into three phenotypes: i) small non-vacuolated cells, ii) intermediate cells with at least one detectable vacuole, and iii) large physaliferous cells with an estimated total vacuole compartment at least the size of the respective nucleus. Each single cell was tracked until performing its first change, namely: a) development (i.e. from a small cell into an intermediate cell), b) cell division into respective phenotypes, c) apoptosis or d) showing no change throughout the whole monitoring (i.e. small cells not dividing or obtaining vacuoles). We excluded cells from the analysis that we could not clearly track (due to escaping the field of view or due to superimposed dividing cells) and that were undergoing cell division either at the beginning (no distinct initial phenotype) or at the end (no distinct terminal phenotype) of the monitoring. p-Values were calculated with Fisher's test for r by c tables using R 2.15.2 [12]. All null hypotheses were two-sided; p-values <0.05 were considered statistically significant. Standard errors of relative frequencies were calculated by the usual moment estimator.

### Ethics Statement

All experimental work was performed according to the Declaration of Helsinki. The study was approved by the ethics committee of the Medical University of Graz (reference EK: 1.8–192 ex 06/07) and written informed consent was obtained from the patient.

## Results

### Morphology and Staining

**Figure 1:**
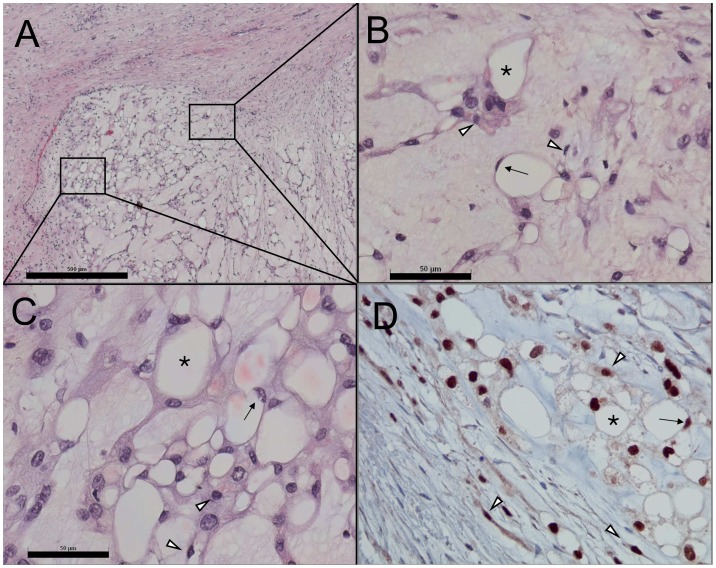
Morphological and immunohistochemical characterization of the chordoma tumor giving rise to MUG-Chor1 cell line . A) Hematoxylin/eosin stained section show lobulated myxoid tumor tissue with cords, strands and nests of tumor cells with pale/eosinophilic to vacuolated cytoplasm. B, C) In detail, the tumor is composed of small cells with eosinophilic cytoplasm and partly spindle cell morphology and large vacuolated/physaliferous tumor cells including “signet ring” shaped cells. D) All cell phenotypes yield the chordoma-specific nuclear staining for brachyury. Arrowheads: small cells; asterisks: large vacuolated/physaliferous cells; arrows: “signet ring” cells. Scale bars: 500 µm (A), 50 µm (B, C); D: original magnification X20.

**Figure 2:**
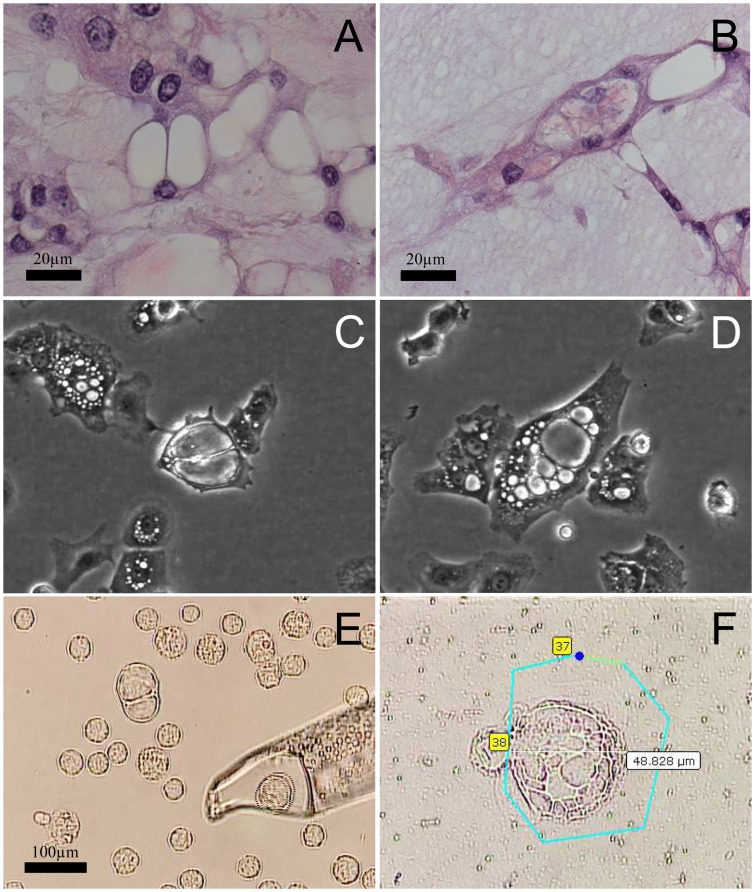
Morphological characteristics of vacuoles in large cells. Large physaliferous cells consisting of equal vacuole content may present with either few very large (A, C, E) or numerous smaller vacuoles (B, D, F). Both morphological phenotypes were detected throughout all samples ranging from tumor tissue (A, B), cell culture (C, D) to sample preparations just before being micromanipulated (E) or microdissected (F) indicating stable characteristics of this chordoma tumor. C, D: original magnification X10; F: original magnification X40.

**Figure 3:**
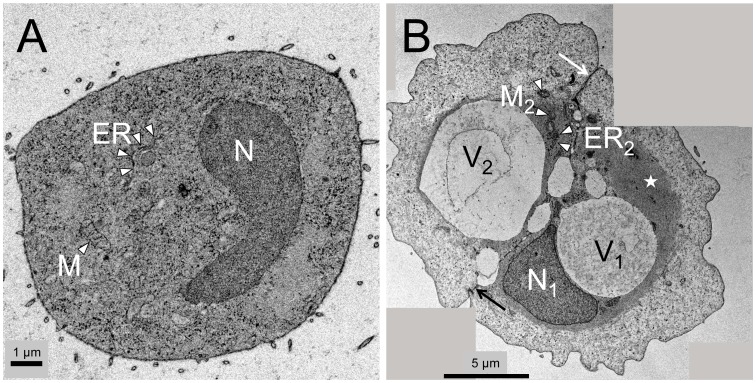
Ultrastructural analysis of small and intermediate cells. A) Small MUG-Chor1 cells show typical ultrastructural features of a diploid cell including nucleus (N), mitochondria (M), and endoplasmic reticulum (ER) in a dense cytoplasm. B) Stitched image of two intermediate cells tightly connected alongside their cell membranes (arrow) present with nucleus (N, only visible in one cell), ER, and mitochondria. Both cells already contain prominent vacuoles (V_1_, V_2_) and show highly organized cytoskeleton in close proximity to the nucleus and vacuoles (asterisk). Scale bars: 1 µm (A), 5 µm (B).

Histological evaluation revealed myxoid, multi-lobulated tumor tissue with cords, strands, and nests of tumor cells with pale/eosinophilic to vacuolated cytoplasm (Figure 1A–C). Immunohistochemical staining of the tissue sections showed cells positive for brachyury, a typical marker for chordoma (Figure 1D). Staining of pan-cytokeratin, EMA, and S100 was also found to be positive as expected for chordoma tissue (data not shown). Microscopic evaluation of MUG-Chor1 cells in culture as well as before microdissection and micromanipulation showed concordant cell morphologies as compared to the tumor tissue (Figure 2). Compared to small MUG-Chor1 cells ultrastructural analysis depicted a high degree of organized cytoplasm in intermediate cells with prominent vacuoles embedded in cytoskeleton structures (Figure 3).

### Morphological Observation of MUG-Chor1 Cells

**Figure 4:**
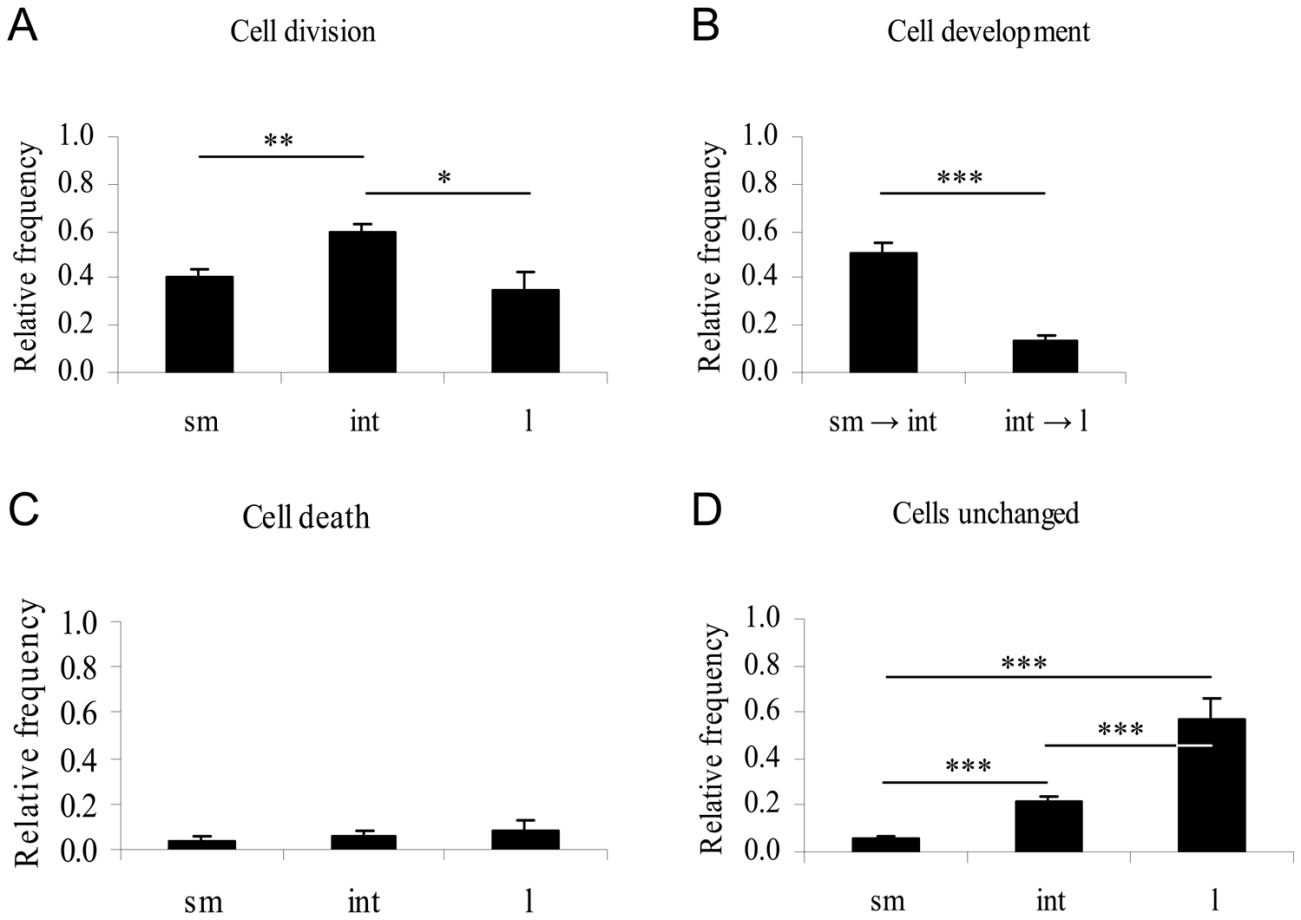
Morphological data analysis of MUG-Chor1 cells. Cells were tracked over a period of seven days with images taken every 30-vacuolated cells (n = 175; sm), intermediate cells containing at least one vacuole (n = 209; int), and large cells containing an estimated total vacuole compartment at least the size of the nucleus (n = 35; l). Cells leaving or entering the monitored areas or undergoing cell division at the very beginning or end of the time lapse were excluded from investigation. A) Intermediate cells significantly divided at a higher rate than small or large cells whereas no difference was seen between small and large cells (p = 0.57). B) Development of small into intermediate cells was significantly higher than intermediate to large cells. No backward-development was detected. C) Cell death rates did not differ significantly. D) The fraction of cells remaining in their phenotype without developing or dividing was found to be highest in large physaliferous cells followed by intermediate and small cells. p-Values as indicated by asterisks: p<0.01 (*); p<0.001 (**), p<0.0001 (***).

**Figure 5:**
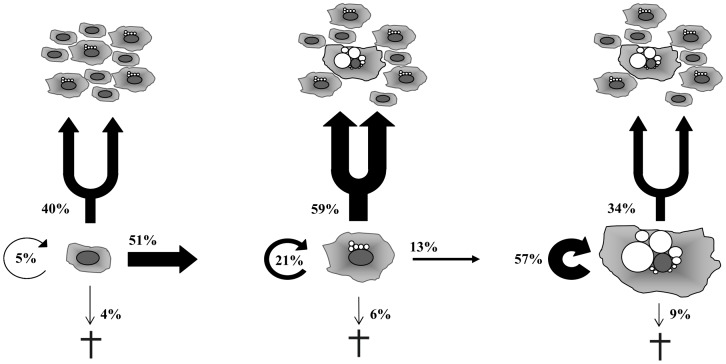
Cell fate of different cell phenotypes suggesting a one-way development. Analysis of the activities of the cells draws a picture of small cells (left) developing into large cells (right) via a mainly proliferative intermediate cell phenotype (middle). As observed in the time lapse experiments the respective cell phenotypes could also emerge through cell division. These cells also fed into the developmental process as depicted (e. g. intermediate cell dividing into one intermediate and one small cell that subsequently develops into an intermediate cell). The developmental process is highly directed as reduction of vacuolization (“backwards development”) was solely observed in dying cells. Due to a proliferation rate comparable to the small cells the large cell phenotype is not representing senescent cells but rather the end of this developmental process. Proportions of cells that undergo proliferation, development, cell death or remain in their original phenotype are given in percentage of the amount of cells allocated to the respective phenotype.

In total we monitored 175 small, 209 intermediate and 35 large physaliferous cells at four different positions (Cell-IQ). A summary of the data and the distinct cell fates are shown in Figure 4 and 5. There is a significant driving force of development from small to intermediate cells compared to intermediate to large physaliferous cells (51% vs. 13%, p<0.0001). The phenotype with the highest proportion of cell divisions is the intermediate phenotype followed by the small non-vacuolated cells (59% vs. 40%, p<0.001) and large phenotype (59% vs. 34%, p<0.01). Interestingly no significant difference could be observed between the cell division rates of the small vs. the large cells (40% vs. 34%, p = 0.57). The highest fraction of cells remaining in their phenotype without cell division could be detected in the large cells followed by the intermediate and small cell phenotype (57% vs. 21% vs. 5%, respectively, p<0.0001). All cell phenotypes show a low rate of apoptosis (4 to 9%) without any statistically significant differences.

### MUG-Chor1 Whole Genome Amplification and Array-CGH

**Figure 6:**
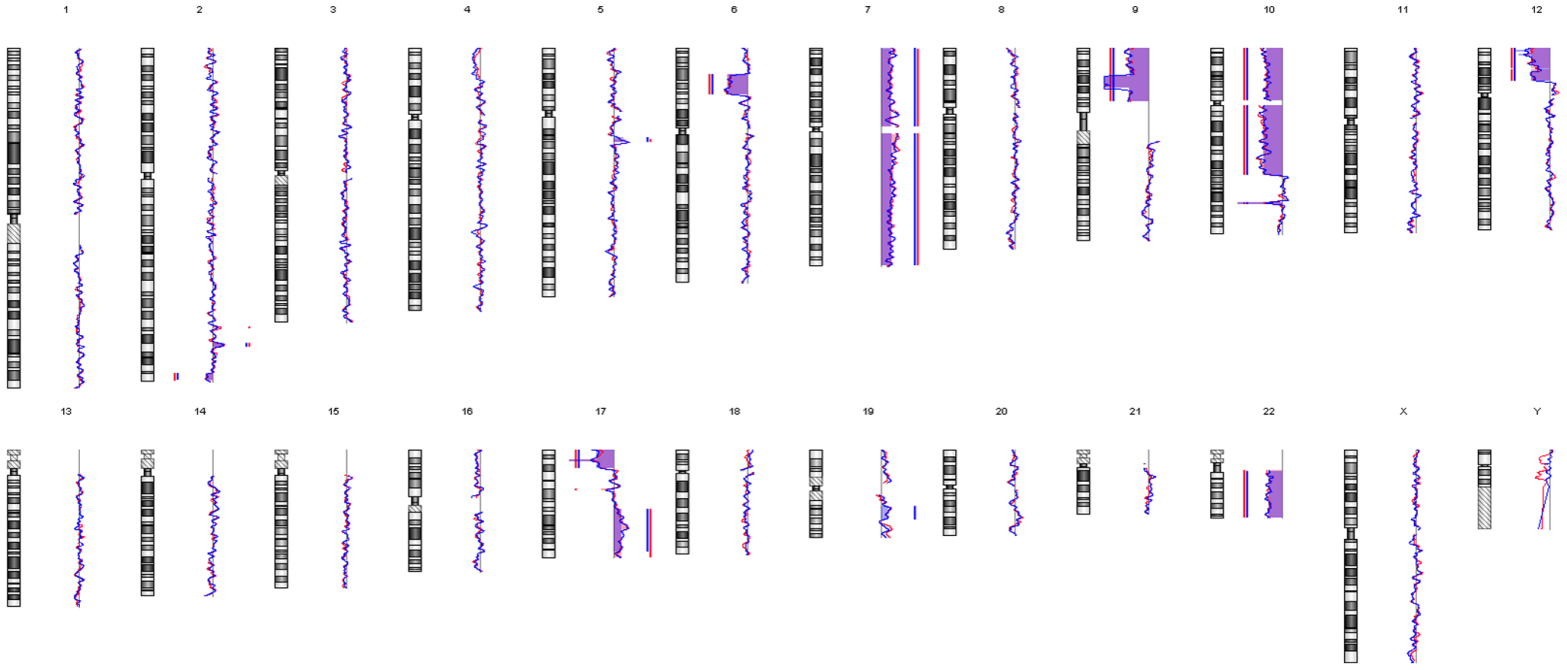
Array-CGH profiles of MUG-Chor1 phenotypes. Comparative genome hybridization of 100 large cells (red line) and small cells (blue line) each yielded identical chromosomal profiles. Both populations show gains at chromosomes 2q, 5q, 7, 17q and losses at 2q, 6p, 9p, 10p, 10q, 12p, 17p and 22. Small gains at chromosomes 2q as well as a small loss at chromosome 17q were detected in the large cell phenotype. This indicates that both morphologically different cell types evolved from a common clonal origin. Bars on the left of the moving average indicate losses of DNA. Bars on the right of the moving average indicate gains of DNA. Both profiles are in line with previously published data [8].

All amplified DNA samples passed the multiplex quality control PCR, demonstrating a sufficient quality for array-CGH analysis. The array-CGH profiles of the two distinct cell phenotypes did not show any obvious differences in their genomic copy number status (Figure 6). In fact, they showed high concordance to each other. In detail, both populations showed gains at chromosomes 2q, 5q, 7, 17q and losses at 2q, 6p, 9p, 10p, 10q, 12p, 17p and 22. Small gains at chromosomes 2q as well as a small loss at chromosome 17q were detected in the large cell phenotype and indicated as a trend in the small cells.

### Low-template Microarray Analysis (MUG-Chor1) and RT-qPCR (MUG-Chor1 and U-CH1)

Affymetrix gene expression analysis and statistical analysis of MUG-Chor1 cells resulted in four significantly differentially expressed genes: *ALG11*, *UCHL3*, *TMEM144* and *PPP2CB* (Table S1). The gene set analysis, GlobalTest, in contrast, produced 398 significant GO terms (Table S2) and 118 significant curated gene sets (Table S3).

RT-qPCR analysis of MUG-Chor1 samples confirmed the gene expression data except for *TMEM144*. Cells of large phenotype showed up-regulation in *ALG11* (695-fold, p = 1.4•10^−4^), *PPP2CB* (18.6-fold, p = 0.0016), and *UCHL3* (18.7-fold, p = 1.9•10^−5^) but down-regulation of *TMEM144* (2.4-fold, p = 0.0061). Expression levels of *T*, *KRT8*, *TGFα*, and *VIM* confirmed the high expression seen in the microarray data with *VIM* showing highest overall expression (Table 1).

We were able to confirm the UCH-L3 overexpression (fold change  = 3.70; p = 0.000226) in the large U-CH1 physaliferous cells. ALG11 (fold change  = 10.58; p = 0.0297) and PPP2CB (fold change  = 4.90; p = 0.0266) showed the same trend as seen in the MUG-Chor1 cells but were not statistically significant (cut-off for multiple testing: p<0.0127). TMEM144 data could not be confirmed in U-CH1 cells (fold change  = 2.11; p = 0.257).

## Discussion

Chordoma tumors are distinguished from other tumors by their heterogeneity with respect to various cell phenotypes in chordoma tissues as well as in chordoma cell lines such as MUG-Chor1 and U-CH1. In this study, we describe chordoma cell characteristics (MUG-Chor1) showing a highly directed development from small non-vacuolated cells to large physaliferous cells via a continuum of intermediate cells. Furthermore, intermediate cells were identified to be the predominant proliferating phenotype. We demonstrate that the different phenotypes in MUG-Chor1 cells share the same copy number variations on the genomic level. Hence, these cells are derived from a common clonal origin and do not represent distinct subpopulations, thus suggesting non-genomic origins of their morphological variation. By cDNA microarray analysis of the two extreme MUG-Chor1 cell phenotypes (small and large physaliferous cells, respectively) we identified four differentially expressed genes, namely *ALG11*, *PPP2CB, TMEM144* and *UCHL3*. All four genes were validated by RT-qPCR in MUG-Chor1 cells. Most important, differential expression of *UCHL3*, a gene involved in cell differentiation, was confirmed in the second chordoma cell line U-CH1. Expression pattern of *ALG11* (protein glycosylation) and PPP2CB (cell cycle control, motility, proliferation) showed the same trends in U-CH1 cells. *TMEM144,* belonging to an orphan 10-transmembrane family receptor of unknown activity, could not be confirmed in U-CH1 cells [22].

In chordoma tumors the driving force for tumor growth, i.e. the proliferative fraction was not yet fully understood [7]. Ultrastructural analysis of chordoma tissue and *in vitro* cultured cells suggested the small cell fraction to be the only proliferating cell phenotype [5], [23], [24]. However, by analyzing MUG-Chor1 cells via time lapse monitoring, we clearly identified the intermediate cells to be the most proliferating phenotype. Surprisingly, the monitoring revealed that the large physaliferous cells were as well capable of proliferation. Moreover, their proliferation rate is as high as for the small cells. The finding of highly organized cytoskeleton surrounding the vacuoles in the intermediate cells seen in ultrastructural analysis suggests a role in MUG-Chor1 intermediate and large cell development.

We confirm that physaliferous cells develop from small non-vacuolated cells through an intermediate state of vacuolization as suggested by others [3]. Interestingly in very rare cases we observed cells reducing its grade of vacuolization, which on first sight may appear to be a backward-development to a prior phenotype (e. g. from intermediate to small cell phenotype). If these cells, however, were traced for a longer period of time, they were found to form apoptotic bodies and died. Thus, we conclude chordoma cells run through a one-way development from the small to the large cell phenotype.

Array-CGH did not reveal any relevant copy number variations between the two MUG-Chor1 cell phenotypes; in fact quite the contrary was detected. Both phenotypes were to a high extent similar which confirms high technical reproducibility of array-CGH from as few as 100 cells. In general the overall aberrations are in line with the copy number status of the mixed whole cell population as we reported previously [8]. The four differentially expressed genes identified in MUG-Chor1 cells (*ALG11*, *PPP2CB*, *TMEM144*, *and UCHL3*) are located at regions without copy number variations.

Microarray expression analysis also showed high concordance between the small cell and large physaliferous cell phenotype suggesting modest biological differences.

*UCHL3* is up-regulated in large MUG-Chor1 (18.7-fold) and U-CH1 (3.7-fold) physaliferous cells suggesting an involvement of the ubiquitin system. The ubiquitin system has been implicated in numerous cellular processes, including protein quality control, cell cycle, membrane protein internalization, signal transduction, apoptosis, and cell proliferation [38], [39]. The removal of covalently attached ubiquitin from the target protein is catalyzed by deubiquitinating enzymes (DUBs). One subfamily of DUBs is the group of ubiquitin C-terminal hydrolases (UCHs). UCHs are able to hydrolyze ubiquitin precursor proteins and remove small adducts from ubiquitin *in vitro* [40] and *in vivo* [41]. *UCHL3*, located on chromosome 13, is expressed in various tissues [42] and was described to recognize and hydrolyze isopeptide bonds at the C-terminal glycine linked to ubiquitin and Nedd8 [43]. Kim *et al.* showed *UCHL3* to interact with Smad1 which is involved in osteoblast differentiation [44], [45]. In mice, Smad1 expression was not only involved in osteogenesis but also in chondrocyte differentiation [46]. Therefore we conclude, that *UCHL3* up-regulation in large physaliferous chordoma cells reflects a process of differentiation from small cells to large physaliferous cells.

The most remarkable difference in MUG-Chor1 cells concern *ALG11* gene expression showing a 695-fold increase in the large physaliferous cell phenotype. *ALG11* is an evolutionary conserved mannosyltransferase within eukaryotes involved in glycosylation of proteins catalyzing the transfer of two mannose sugar units to Man_3_GlcNAc_2_-PP-dolichol, which yields Man_5_GlcNAc_2_-PP-dolichol [25], [26]. *ALG11* is also known to cause severe human metabolic disease if mutated [27]. In glycosylation these are the last two elongation steps taking place in the cytoplasm before the oligosaccharide/dolichol is transferred into the lumen of the endoplasmatic reticulum to be further elongated. Although only described in the context of glycosylation, we hypothesize that *ALG11* could be involved in biosynthesis of glucosaminoglycans/aggrecan that represent a key component of the extracellular matrix of chorda dorsalis, nucleosus pulposus, and classical chordoma [28]. Although being not statistically significant we could see a similar trend in large U-CH1 cells (10.6-fold; p = 0.03; cut-off for multiple testing: p<0.0127).

The gene *PPP2CB* is 18.6-fold up-regulated in large physaliferous MUG-Chor1 cells. It encodes for the catalytic subunit PP2A_c_ beta (β) of the protein phosphatase 2A (PP2A) showing a 97% protein sequence identity to its isoform PP2A_c_ alpha (α, *PPP2CA*) [29]. PP2A is a major serine/threonine phosphatase, consisting of a core dimer of a regulatory subunit A and the catalytic subunit C. A variable third regulatory B subunit can be associated to the core dimer, thereby steering the substrate specificity of the PP2A complex [30]. The PP2A holoenzyme family is involved in several biological processes including cell growth, multiple signaling pathways, differentiation and cell motility [30]–[32]. We observed a decreased expression of the PP2A_c_ catalytic β subtype in the small cell compared to the large cell phenotype. While PP2A_c_ α is well described in literature, only little information of the deregulated PP2A_c_ β is available. In prostate cancer, Prowatke and coworker re-analyzed previously published genomic- [33], [34] and expression-profiling studies for identifying candidate genes relevant for prostate tumor prognosis and progression [35]. They found *PPP2CB* to be deleted in 23.5% of 145 primary prostate cancers analyzed by means of comparative genomic hybridization [33]. Another five of 16 prostate cancer tumors showed *PPP2CB* deletion identified by array-CGH [34]. Despite being down-regulated in primary prostate cancers, *PPP2CB* did not correlate with clinico-pathological factors [35]. Similar to Prowatke *et al.* we also found *PPP2CB* to be differently expressed but on the contrary our data clearly show *PPP2CB* expression correlating with MUG-Chor1 cell morphology. More recent data suggested PP2A_c_ β to be involved in a novel cell cycle regulatory pathway via interaction with *FHL1B* [36]. The latter contains a Lin11/Isl-1/Mec-3 (LIM) domain thereby providing a modular protein-binding interface. Through this domain, *FHL1B* might function as a biosensor mediating communication between cytosolic and nuclear compartments [37], possibly linking PP2A_c_ β to a cell cycle regulatory pathway [36]. However, our data do not provide evidence for *PPP2CB* being involved in cell cycle and apoptosis of MUG-Chor1 cells as we detected no differences in cell division and apoptosis rates in between the two phenotypes. Similar to the data seen in *ALG11*, *PPP2CB* is not significantly overexpressed in the large U-CH1 cell phenotype. However, there seems to be a trend towards up-regulation in the large cells (4.9-fold; p = 0.026; cut-off for multiple testing: p<0.0127).

In microarray analysis *TMEM144* was found to be up-regulated in large physaliferous MUG-Chor1 cells. In contrast, RT-qPCR yielded a 2.4-fold (p = 0.0061) down-regulation in the large cells compared to the small cell phenotype. This inconsistency clears away when comparing the expression array to the TaqMan assay data involved in *TMEM144* analysis: the Affymetrix GeneChip Human 1.0 ST array covers 39 sites throughout the *TMEM144* locus, whereas the primer of the recommended TaqMan assay (Hs00938021_m1) covers exon 11, a region represented with two microarray spots. The microarray data yield a 2.74-fold up-regulation in the large cells across all 39 spots. However, spots 30 and 31, which cover exon 11, yield lower signals (data not shown). However, Prentice and colleagues linked *TMEM144* to the regulation of kisspeptin [22] that itself seems to be involved in cancer by suppressing metastasis due to inhibition of cancer cell motility [47]. *TMEM144* is not differentially expressed in the U-CH1 cell phenotypes.

## Conclusions

We identified a directed development from small to large physaliferous cells via intermediate cells being the main proliferating cell phenotype. We confirm previous findings showing the small cells to be proliferating. Interestingly, we also found the large physaliferous cells to be as proliferative as the small cells. We identified *UCHL3* to be a key player in chordoma cell development due to its up-regulation in the large physaliferous cell phenotype of both tested chordoma cell lines (MUG-Chor1 and U-CH1). We found *ALG11* and *PPP2CB* to be up- and *TMEM144* to be differentially regulated in large physaliferous MUG-Chor1 cells, hence being putative effector genes for chordoma cell development.

## Supporting Information

Table S1 **Affymetrix gene expression analysis and statistical analysis.** This file provides details of the four genes (*ALG11*, *UCHL3*, *TMEM144* and *PPP2CB*) found to be significantly differentially expressed in MUG-Chor1 cells based on Affymetrix gene expression and subsequent statistical analysis.
(XLS)

Table S2 **GlobalTest.** This file lists the 398 GO terms being associated with the expression data of MUG-Chor1 cells obtained by GlobalTest (gene set analysis).
(XLS)

Table S3 **MSigDB data set.** This file lists the 118 gene sets being associated with the expression data of MUG-Chor1 cells identified by means of MSigDB.
(XLS)

Video S1 **MUG-Chor1 cell monitoring.** This Cell-IQ video shows MUG-Chor1 cell line cells (passage 30) monitored in a 6-well plate over a period of seven days at an image rate of one image per 30 min. Four different positions were evaluated by video analysis to assess the MUG-Chor1 cell line characteristics regarding cell division and development. From the time lapse experiments we were able to identify small, intermediate and large physaliferous cells to undergo cell division. Furthermore, we monitored cells exhibiting diverse vacuole activities such as producing considerable amounts of vacuoles (i.e. intermediate cells developing into large physaliferous cells) or vacuole fusion resulting in “signet ring”-shaped cells. The latter are very similar to the cells in the tumor tissue (see Figure 1). The video was adapted using AWS Video Converter version 8.3.2.533. Original magnification: 10x objective.
(AVI)

## References

[1] Chugh R, Tawbi H, Lucas DR, Biermann JS, Schuetze SM, et al (2007) Chordoma: the nonsarcoma primary bone tumor. Oncologist 12: 1344–1350. 18055855 10.1634/theoncologist.12-11-1344

[2] Fletcher CD, McKee PH (1985) Immunohistochemistry and histogenesis of extraskeletal myxoid chondrosarcoma. J Pathol 147: 67–68. 4045599 10.1002/path.1711470109

[3] Erlandson RA, Tandler B, Lieberman PH, Higinbotham NL (1968) Ultrastructure of human chordoma. Cancer Res 28: 2115–2125. 5696940

[4] Gui X, Siddiqui NH, Guo M (2004) Physaliphorous cells in chordoma. Arch Pathol Lab Med 128: 1457–1458. 15578899 10.5858/2004-128-1457-PCIC

[5] Murad TM, Murthy MS (1970) Ultrastructure of a chordoma. Cancer 25: 1204–1215. 5443740 10.1002/1097-0142(197005)25:5<1204::aid-cncr2820250528>3.0.co;2-6

[6] Chabner B, Amrein P, Druker B, Michaelson M, Mitsiades C, et al.. (2006) Chemotherapy of neoplastic diseases. In: Brunton L, editor. Goodman & Gilman's The pharmacological basis of therapeutics. 11 ed. United States: McGraw-Hill. pp. 1315–1404.

[7] Brüderlein S, Sommer JB, Meltzer PS, Li S, Osada T, et al.. (2010) Molecular characterization of putative chordoma cell lines. Sarcoma: 630129. 10.1155/2010/630129 PMC3022207 21253487

[8] Rinner B, Froehlich EV, Buerger K, Knausz H, Lohberger B, et al (2012) Establishment and detailed functional and molecular genetic characterisation of a novel sacral chordoma cell line, MUG-Chor1. Int J Oncol 40: 443–451. 22002331 10.3892/ijo.2011.1235

[9] Kroneis T, Geigl JB, El-Heliebi A, Auer M, Ulz P, et al (2011) Combined molecular genetic and cytogenetic analysis from single cells after isothermal whole-genome amplification. Clin Chem 57: 1032–1041. 21558453 10.1373/clinchem.2011.162131 PMC3356848

[10] Geigl JB, Speicher MR (2007) Single-cell isolation from cell suspensions and whole genome amplification from single cells to provide templates for CGH analysis. Nat Protoc 2: 3173–3184. 18079717 10.1038/nprot.2007.476

[11] van Beers EH, Joosse SA, Ligtenberg MJ, Fles R, Hogervorst FB, et al (2006) A multiplex PCR predictor for aCGH success of FFPE samples. Br J Cancer 94: 333–337. 16333309 10.1038/sj.bjc.6602889 PMC2361127

[12] R Development Core Team (2012) R: A language and environment for statistical computing. Vienna, Austria: R Foundation for Statistical Computing.

[13] Irizarry RA, Bolstad BM, Collin F, Cope LM, Hobbs B, et al (2003) Summaries of Affymetrix GeneChip probe level data. Nucleic Acids Res 31: e15. 12582260 10.1093/nar/gng015 PMC150247

[14] Irizarry RA, Hobbs B, Collin F, Beazer-Barclay YD, Antonellis KJ, et al (2003) Exploration, normalization, and summaries of high density oligonucleotide array probe level data. Biostatistics 4: 249–264. 12925520 10.1093/biostatistics/4.2.249

[15] Gautier L, Cope L, Bolstad BM, Irizarry RA (2004) affy–analysis of Affymetrix GeneChip data at the probe level. Bioinformatics 20: 307–315. 14960456 10.1093/bioinformatics/btg405

[16] Scholtens D, von Heydebreck A (2005) Analysis of Differential Gene Expression Studies. In: Gentleman R, Carey V, Huber W, Irizarry R, Dudoit S, editors. Bioinformatics and Computational Biology Solutions Using R and Bioconductor. New York: Springer. pp. 229–248.

[17] Smyth GK (2005) Limma: Linear Models for Microarray Data. In: Gentleman R, Carey V, Huber W, Irizarry R, Dudoit S, editors. Bioinformatics and Computational Biology Solutions Using R and Bioconductor. New York: Springer. pp. 397–420.

[18] Benjamini Y, Hochberg Y (1995) Controlling the false discovery rate - a practical and powerful approach to multiple testing. J R Stat Soc Series B Stat Methodol 57: 289–300.

[19] Goeman JJ, van de Geer SA, de Kort F, van Houwelingen HC (2004) A global test for groups of genes: testing association with a clinical outcome. Bioinformatics 20: 93–99. 14693814 10.1093/bioinformatics/btg382

[20] Subramanian A, Tamayo P, Mootha VK, Mukherjee S, Ebert BL, et al (2005) Gene set enrichment analysis: a knowledge-based approach for interpreting genome-wide expression profiles. Proc Natl Acad Sci U S A 102: 15545–15550. 16199517 10.1073/pnas.0506580102 PMC1239896

[21] Burns MJ, Nixon GJ, Foy CA, Harris N (2005) Standardisation of data from real-time quantitative PCR methods - evaluation of outliers and comparison of calibration curves. BMC Biotechnol 5: 31. 16336641 10.1186/1472-6750-5-31 PMC1326201

[22] Prentice LM, d'Anglemont de Tassigny X, McKinney S, Ruiz de Algara T, Yap D, et al (2011) The testosterone-dependent and independent transcriptional networks in the hypothalamus of Gpr54 and Kiss1 knockout male mice are not fully equivalent. BMC Genomics 12: 209. 21527035 10.1186/1471-2164-12-209 PMC3111392

[23] Fu YS, Pritchett PS (1975) Tissue culture study of a sacrococcygeal chordoma with further ultrastructural study. Acta Neuropathol 32: 225–233. 1101623 10.1007/BF00696571

[24] Horten BC, Montague SR (1976) In vitro characteristics of a sacrococcygeal chordoma maintained in tissue and organ culture systems. Acta Neuropathol 35: 13–25. 1274529 10.1007/BF00688940

[25] Cipollo JF, Trimble RB, Chi JH, Yan Q, Dean N (2001) The yeast ALG11 gene specifies addition of the terminal alpha 1,2-Man to the Man5GlcNAc2-PP-dolichol N-glycosylation intermediate formed on the cytosolic side of the endoplasmic reticulum. J Biol Chem 276: 21828–21840. 11278778 10.1074/jbc.M010896200

[26] Umeda K, Yoko-o T, Nakayama K, Suzuki T, Jigami Y (2000) Schizosaccharomyces pombe gmd3(+)/alg11(+) is a functional homologue of Saccharomyces cerevisiae ALG11 which is involved in N-linked oligosaccharide synthesis. Yeast 16: 1261–1271. 11015724 10.1002/1097-0061(200010)16:14<1261::AID-YEA620>3.0.CO;2-9

[27] Rind N, Schmeiser V, Thiel C, Absmanner B, Lubbehusen J, et al (2010) A severe human metabolic disease caused by deficiency of the endoplasmatic mannosyltransferase hALG11 leads to congenital disorder of glycosylation-Ip. Hum Mol Genet 19: 1413–1424. 20080937 10.1093/hmg/ddq016

[28] Gottschalk D, Fehn M, Patt S, Saeger W, Kirchner T, et al (2001) Matrix gene expression analysis and cellular phenotyping in chordoma reveals focal differentiation pattern of neoplastic cells mimicking nucleus pulposus development. Am J Pathol 158: 1571–1578. 11337353 10.1016/S0002-9440(10)64111-9 PMC1891956

[29] Arino J, Woon CW, Brautigan DL, Miller TB Jr, Johnson GL (1988) Human liver phosphatase 2A: cDNA and amino acid sequence of two catalytic subunit isotypes. Proc Natl Acad Sci U S A 85: 4252–4256. 2837763 10.1073/pnas.85.12.4252 PMC280405

[30] Janssens V, Goris J (2001) Protein phosphatase 2A: a highly regulated family of serine/threonine phosphatases implicated in cell growth and signalling. Biochem J 353: 417–439. 11171037 10.1042/0264-6021:3530417 PMC1221586

[31] Basu S (2011) PP2A in the regulation of cell motility and invasion. Curr Protein Pept Sci 12: 3–11. 21190527 10.2174/138920311795659443

[32] Götz J, Probst A, Ehler E, Hemmings B, Kues W (1998) Delayed embryonic lethality in mice lacking protein phosphatase 2A catalytic subunit Calpha. Proc Natl Acad Sci U S A 95: 12370–12375. 9770493 10.1073/pnas.95.21.12370 PMC22838

[33] Alers JC, Krijtenburg PJ, Vis AN, Hoedemaeker RF, Wildhagen MF, et al (2001) Molecular cytogenetic analysis of prostatic adenocarcinomas from screening studies: early cancers may contain aggressive genetic features. Am J Pathol 158: 399–406. 11159178 10.1016/s0002-9440(10)63983-1 PMC1850287

[34] Paris PL, Albertson DG, Alers JC, Andaya A, Carroll P, et al (2003) High-resolution analysis of paraffin-embedded and formalin-fixed prostate tumors using comparative genomic hybridization to genomic microarrays. Am J Pathol 162: 763–770. 12598311 10.1016/S0002-9440(10)63873-4 PMC1868117

[35] Prowatke I, Devens F, Benner A, Grone EF, Mertens D, et al (2007) Expression analysis of imbalanced genes in prostate carcinoma using tissue microarrays. Br J Cancer 96: 82–88. 17146477 10.1038/sj.bjc.6603490 PMC2360197

[36] Wong CH, Fung YW, Ng EK, Lee SM, Waye MM, et al (2010) LIM domain protein FHL1B interacts with PP2A catalytic beta subunit–a novel cell cycle regulatory pathway. FEBS Lett 584: 4511–4516. 20969868 10.1016/j.febslet.2010.10.022

[37] Kadrmas JL, Beckerle MC (2004) The LIM domain: from the cytoskeleton to the nucleus. Nat Rev Mol Cell Biol 5: 920–931. 15520811 10.1038/nrm1499

[38] Amerik AY, Hochstrasser M (2004) Mechanism and function of deubiquitinating enzymes. Biochim Biophys Acta 1695: 189–207. 15571815 10.1016/j.bbamcr.2004.10.003

[39] Weissman AM (2001) Themes and variations on ubiquitylation. Nat Rev Mol Cell Biol 2: 169–178. 11265246 10.1038/35056563

[40] Larsen CN, Krantz BA, Wilkinson KD (1998) Substrate specificity of deubiquitinating enzymes: ubiquitin C-terminal hydrolases. Biochemistry 37: 3358–3368. 9521656 10.1021/bi972274d

[41] Setsuie R, Suzuki M, Tsuchiya Y, Wada K (2010) Skeletal muscles of Uchl3 knockout mice show polyubiquitinated protein accumulation and stress responses. Neurochem Int 56: 911–918. 20380862 10.1016/j.neuint.2010.03.021

[42] Kurihara LJ, Semenova E, Levorse JM, Tilghman SM (2000) Expression and functional analysis of Uch-L3 during mouse development. Mol Cell Biol 20: 2498–2504. 10713173 10.1128/mcb.20.7.2498-2504.2000 PMC85452

[43] Hemelaar J, Borodovsky A, Kessler BM, Reverter D, Cook J, et al (2004) Specific and covalent targeting of conjugating and deconjugating enzymes of ubiquitin-like proteins. Mol Cell Biol 24: 84–95. 14673145 10.1128/MCB.24.1.84-95.2004 PMC303361

[44] Kim BG, Lee JH, Ahn JM, Park SK, Cho JH, et al (2009) 'Two-stage double-technique hybrid (TSDTH)' identification strategy for the analysis of BMP2-induced transdifferentiation of premyoblast C2C12 cells to osteoblast. J Proteome Res 8: 4441–4454. 19655815 10.1021/pr900231a

[45] Kim JY, Lee JM, Cho JY (2011) Ubiquitin C-terminal hydrolase-L3 regulates Smad1 ubiquitination and osteoblast differentiation. FEBS Lett 585: 1121–1126. 21453705 10.1016/j.febslet.2011.03.053

[46] Flanders KC, Kim ES, Roberts AB (2001) Immunohistochemical expression of Smads 1–6 in the 15-day gestation mouse embryo: signaling by BMPs and TGF-betas. Dev Dyn 220: 141–154. 11169847 10.1002/1097-0177(2000)9999:9999<::AID-DVDY1096>3.0.CO;2-4

[47] Cho SG, Li D, Tan K, Siwko SK, Liu M (2012) KiSS1 and its G-protein-coupled receptor GPR54 in cancer development and metastasis. Cancer Metastasis Rev 31: 585–591. 22692479 10.1007/s10555-012-9367-7

